# High Passage MIN6 Cells Have Impaired Insulin Secretion with Impaired Glucose and Lipid Oxidation

**DOI:** 10.1371/journal.pone.0040868

**Published:** 2012-07-13

**Authors:** Kim Cheng, Viviane Delghingaro-Augusto, Christopher J. Nolan, Nigel Turner, Nicole Hallahan, Sofianos Andrikopoulos, Jenny E. Gunton

**Affiliations:** 1 Diabetes and Transcription Factors Group, Garvan Institute of Medical Research (GIMR), Sydney, NSW, Australia; 2 Medical School, The Australian National University, Canberra, ACT, Australia; 3 Diabetes and Obesity Program, Garvan Institute of Medical Research (GIMR), Sydney, NSW, Australia; 4 Department of Medicine (AH/NH), University of Melbourne, Melbourne, Victoria, Australia; 5 Faculty of Medicine, University of Sydney, Sydney, NSW, Australia; 6 St. Vincent’s Clinical School, University of New South Wales, Sydney, NSW, Australia; 7 Department of Diabetes and Endocrinology, Westmead Hospital, Sydney, NSW, Australia; Universita Magna-Graecia di Catanzaro, Italy

## Abstract

Type 2 diabetes is a metabolic disorder characterized by the inability of beta-cells to secrete enough insulin to maintain glucose homeostasis. MIN6 cells secrete insulin in response to glucose and other secretagogues, but high passage (HP) MIN6 cells lose their ability to secrete insulin in response to glucose. We hypothesized that metabolism of glucose and lipids were defective in HP MIN6 cells causing impaired glucose stimulated insulin secretion (GSIS). HP MIN6 cells had no first phase and impaired second phase GSIS indicative of global functional impairment. This was coupled with a markedly reduced ATP content at basal and glucose stimulated states. Glucose uptake and oxidation were higher at basal glucose but ATP content failed to increase with glucose. HP MIN6 cells had decreased basal lipid oxidation. This was accompanied by reduced expressions of *Glut1, Gck, Pfk, Srebp1c*, *Ucp2, Sirt3, Nampt*. MIN6 cells represent an important model of beta cells which, as passage numbers increased lost first phase but retained partial second phase GSIS, similar to patients early in type 2 diabetes onset. We believe a number of gene expression changes occurred to produce this defect, with emphasis on *Sirt3* and *Nampt*, two genes that have been implicated in maintenance of glucose homeostasis.

## Introduction

Type 2 diabetes (T2D) is characterized by the inability of beta-cells to secrete enough insulin to maintain glucose homeostasis, usually accompanied by insulin resistance: impaired action of insulin on target tissues. Beta-cells secrete insulin in a biphasic manner. The first phase occurs rapidly, commonly defined as <10 minutes after glucose stimulation. In the whole body, insulin secretion normally peaks at 1–2 minutes [1]. Second phase secretion is more gradual and long lasting, typically reaching a plateau at 25–30 minutes [1].

The classic pathway of insulin secretion is generally accepted and begins with glucose entry into beta-cells, and a rise in the ATP:ADP ratio as glucose is metabolized via glycolysis and oxidative phosphorylation [2]–[4]. This closes ATP-dependent K^+^ channels (K_ATP_) and depolarizes the plasma membrane [5], [6]. This then opens the voltage-gated calcium channels, allowing Ca^2+^ to enter the cells, leading to subsequent insulin exocytosis [7]. Interestingly, patients early in the course of T2D lose first phase but often retain second phase insulin secretion [8]. Impairment of first phase secretion is a predictor of future type 1 and T2D risk [9]. Many additional mechanisms regulate insulin secretion including the rate of anapleurosis and changes in glutamate, GTP, and GDP levels (all of which are involved in the amplifying pathway) [4].

The MIN6 cell line was derived from a mouse insulinoma and is one of a few cell lines that display characteristics of pancreatic beta-cells, including insulin secretion in response to glucose and other secretagogues [10], [11]. It has been reported that MIN6 cells with high passage (HP) numbers lose their ability to secrete insulin [12]–[14]. HP MIN6 cells have gene expression changes, including downregulation of genes such as *phospholipase D1* and *cholecystokinin* [14].

HP and low passage (LP) MIN6 cells also differ at the protein level, with HP cells having lowered expression of some proteins that are involved with correct protein folding in the ER and antioxidant enzymes for handling of ROS [12]. This is in contrast to studies comparing glucose responsive and unresponsive sublines of LP MIN6 cells, with one study showing no change in Glut2 expression [15] while another study showing barely detectable Glut2 expression, even in their glucose responsive MIN6 cell line [16].

These studies showed changes in gene and protein expression, however metabolic changes have not been profiled with a panel of functional assays in detail before. We sought to identify changes associated with impaired insulin secretion in HP cells. HP cells lost first phase insulin secretion and had an overall impairment in GSIS. This was coupled with a markedly reduced intracellular ATP in HP cells, with decreased glucose uptake, glucose oxidation and basal lipid oxidation. HP MIN6 cells had significantly reduced expression of glycolytic genes and genes involved with lipid handling, including *Srebp1c*. This was accompanied by a decrease in *Sirt3* and *Nampt* gene expression.

## Materials and Methods

### Cell Culture

We obtained MIN6 cells from Dr. Ross Laybutt (Garvan Institute of Medical Research) [17] (originally from Dr Miyazaki [11]) and cells were routinely maintained in Dulbecco’s modified Eagle’s medium (DMEM) containing 25 mM glucose, supplemented with 10% fetal calf serum, 2 mM L-glutamine, 25 mM Hepes, and 285 µM 2-mercaptoethanol. Subculture and maintenance were performed as previously described [13]. MIN6 cells presented in this study were at passages 30–40 (low passage, LP) or passages 60–70 (high passage, HP). We compared earlier passage cells (P26–27) and they did not differ in normal GSIS from P30–40 (data not shown). All assays used MIN6 grown to 70–80% confluence unless otherwise stated.

### Electron Microscopy

Electron microscopy samples were fixed for 30 minutes in cacodylate buffered 2% glutaraldehyde. Samples were post-fixed for 90 minutes in 2% osmium tetroxide and then enbloc stained with 2% aqueous uranyl acetate (30 minutes). Samples were dehydrated through a series of ethanols and infiltrated with TAAB epoxy resin. The blocks were set at 80°C overnight. Methylene blue/Azure II stained thick sections were used to identify suitable areas of the blocks which were cut at 100 nm. These sections were collected on Cu/Pd grids, stained with Reynolds lead citrate and viewed on a JEOL 1011 electron microscope with images captured using a MegaView III digital camera and AnalySIS software package.

### Cell Proliferation

Cell proliferation was examined using the FITC BrdU Flow Kit (BD Pharmingen, San Diego, CA, USA). MIN6 cells were pulsed with 10 µl/ml of BrdU solution (1 mM BrdU in PBS) for 40 minutes in MIN6 media at 37°C with 5% CO_2_. Cells were dislodged by trypsin. BrdU and 7AAD staining was performed as per the protocol. Flow cytometric data was acquired using a FACS Calibur (BD Biosciences, San Jose, CA, USA) and FlowJo software (Tree Star).

### Insulin Secretion Assays

Media used for insulin secretion was serum free DMEM with no glucose, supplemented with 2 mM L-glutamine and 25 mM Hepes, pH 7.4. Glucose was added to prepare basal (1 mM) and other glucose concentrations (3.3 mM, 5.5 mM, 11 mM, and 25 mM) and warmed to 37°C prior to use. MIN6 cells were washed twice with PBS and placed in basal serum free media for 2 hours, washed with fresh basal media and placed in the stated media for 15 minutes. Media was then replaced with media containing higher concentrations of glucose for 15 minutes. 15 minutes was the minimum time necessary for the cells and culture media to reach 37°C (approx. 5 minutes) and insulin secretion (approx. 10 minutes) to occur. After completion of the incubations, the cells were lysed with acid/ethanol for measurement of total insulin content as previously reported [18], [19]. Insulin secretion with addition of 1 mM pyruvate, 30 mM L-arginine, or 30 mM KCl was performed after an incubation time of 30 minutes. Bromopalmitate (0.0625 µM) was added 2 hours before insulin secretion assays.

For insulin secretion time courses, MIN6 cells were grown in 12-well plates and samples for basal 1 mM insulin secretion was collected as above. After the addition of 25 mM serum free media, 10 µl was removed at each time point (2, 5, 10, 15, 30, 60 minutes) and total insulin collected as described above. Insulin was measured by ELISA (Crystal Chem, Downers Grove, IL, USA).

### Measurement of Intracellular ATP Content

Intracellular ATP content was measured using the ATP Bioluminescence Assay Kit CLS II (Roche, Sydney, NSW, Australia) according to the manufacturer’s instructions. Cells were equilibrated as described above then serum free media was replaced with fresh basal media. Glucose was added to make up to 25 mM at each time point. MIN6 cells were placed on ice, washed twice with ice cold PBS, and lysed. Results were corrected for total protein.

### Glucose Oxidation Assay

MIN6 cells were grown in 25 cm^2^ flasks, washed twice with PBS, and incubated in Krebs buffer (115 mM NaCl, 4.7 mM KCl, 1 mM MgSO_4_, 1.2 mM KH_2_PO_4_, 25 mM NaHCO_3_, 1 mM sodium pyruvate, 10 mM Hepes, pH 7.4) with 1mM glucose for 2 hours. After equilibration, cells were washed with fresh Krebs buffer with 1mM glucose and media replaced with fresh Krebs buffer at 1, 5, 11, or 25 mM glucose with 0.1 µCi/ml of D-[U-^14^C]-glucose (GE Healthcare, Port Washington, NY, USA). Filter paper soaked in 5% KOH was suspended over the cells and the flasks sealed shut. Cells were incubated at 37°C with 5% CO_2_ for 1 hour and the reaction stopped by the addition of perchloric acid. Radioactivity was counted in 4 ml Microscint-20 (Perkin Elmer, Waltham, Massachusetts, USA) using the LS 6500 Scintillation Counter (Beckman Coulter, Brea, CA, USA). Results were corrected for specific activity and total protein.

### Glucose Uptake Assay

MIN6 cells were grown in 6-well plates and equilibrated as per glucose oxidation protocols. After washing, media was replaced with fresh warm Krebs buffer with 1 or 25 mM glucose and 1 µCi/ml 2-deoxy-[1,2-^3^H]-glucose (Perkin Elmer, Waltham, Massachusetts, USA). Cells were incubated at 37°C with 5% CO_2_ for exactly 5 minutes. The reaction was stopped by placing on ice and washing twice with ice cold 5% glucose in PBS. Cells were lysed with 500 µl of RIPA buffer (0.5% sodium deoxycholate, 50 mM Hepes, 1% NP40, 0.1% SDS, pH 7.4) and counted as above. Results were corrected for total protein.

### Lipid Oxidation

MIN6 cells were grown in 25 cm^2^ flasks, washed twice with PBS, and incubated in Krebs buffer plus 0.25% fatty acid free BSA (Sigma-Aldrich, St. Louis, MO, USA) with 1 mM glucose at 37°C with 5% CO_2_ for 2 hours. After equilibration, cells were washed with fresh Krebs+BSA buffer with 1 mM glucose and media replaced with fresh Krebs+BSA buffer at 1 or 25 mM glucose concentrations with 0.125 mM palmitate and 0.25 µCi/ml of [1-^14^C]-palmitic acid (GE Healthcare, Port Washington, NY, USA). Filter paper soaked in 5% KOH was suspended over the cells and the flasks sealed shut. MIN6 cells were incubated at 37°C with 5% CO_2_ for 24 hours and the reaction stopped by the addition of perchloric acid. Radioactivity was measured as above. Results were corrected for total protein.

### Measurement of Lactate

Cells were incubated in serum free media containing 1 or 25 mM glucose for 2 hours and lactate was measured using the BioVision Lactate Assay Kit II (BioVision, Mountain View, Cal, USA) according to manufacturer’s protocol. Results were corrected for total protein.

### Real-time PCR

RNA was isolated as previously described [18] and real-time PCR performed as previously described [18], [19]. Primer sequences are available on request. Every plate included a house-keeping gene (TATA-box binding protein (TBP)) for LP and HP cells.

### Statistical Analysis

For all figures, error bars indicate ± SEM. Unpaired 2-tailed t-tests were used to compare two variables, and ANOVA with post-hoc testing (Bonferoni or Tukey’s) was used for multiple comparisons. A p-value of <0.05 was considered significant.

## Results

### Insulin Secretion

**Figure 1:**
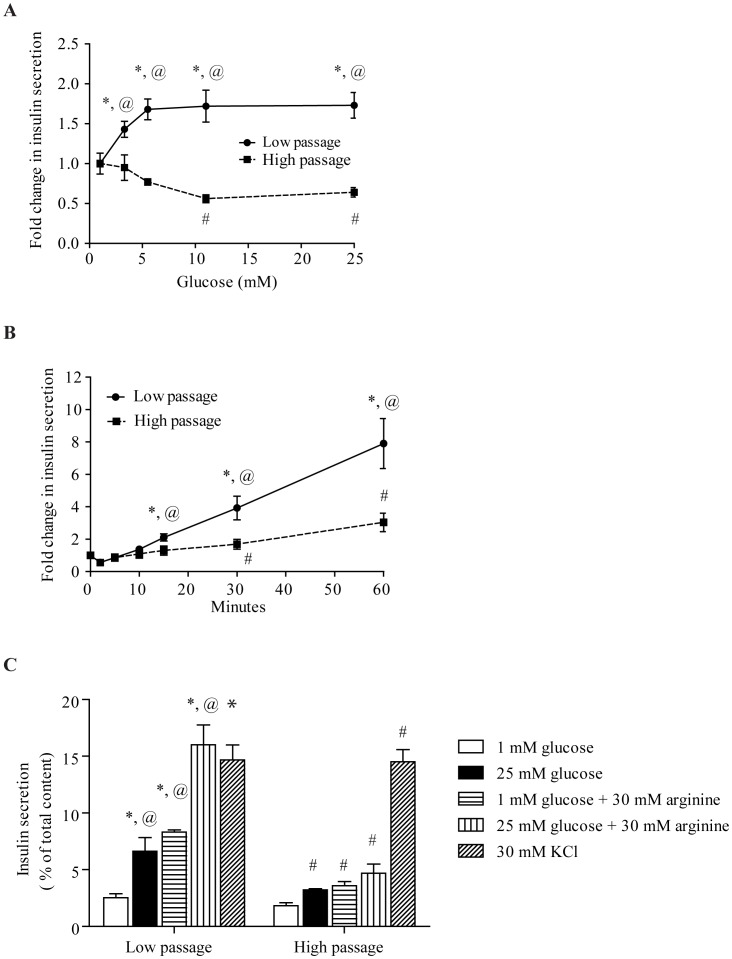
Insulin secretion in LP and HP MIN6 cells. A) LP MIN6 cells responded in a dose dependent manner to glucose but this was not observed in HP MIN6 cells (samples taken at 15 minutes after stimulation, representing first phase insulin secretion). B) A GSIS time course showed that HP MIN6 cells still retained partial second phase insulin secretion, although significantly lower than that observed in LP MIN6 cells. C) After an incubation time of 30 minutes, LP MIN6 cells responded well to the non glucose secretagogues L-arginine and KCl. HP MIN6 cells showed no augmentation in insulin secretion with L-arginine but responded to KCl, with levels comparable to LP MIN6 cells. Error bars are ± SEM and n = 6. * p<0.05 compared to LP MIN6 cells at basal, # p<0.05 compared to HP MIN6 cells at basal, @ p<0.05 LP vs. HP MIN6 cells at their respective glucose concentrations or time points.

Low passage (LP) was defined as passages 30–40 and high passage (HP) as passages 60–70. As shown in Figure 1A, LP cells displayed a dose response in insulin secretion when stimulated with increasing glucose concentrations. This culminated in a ∼1.7-fold increase in insulin secretion with high glucose (25 mM) (p<0.05). This response was not observed in HP cells. There was a trend for higher insulin release at basal glucose (1 mM, p = 0.2, data not shown). Glucose stimulated insulin secretion time courses were studied and as expected, LP MIN6 cells secreted increasing amounts of insulin over time (Figure 1B). Interestingly, HP cells eventually responded to glucose at 60 minutes, however this was still significantly less than LP MIN6 cells (p<0.05). Thus, HP cells had an overall impairment in GSIS, with a more severe effect on first phase secretion.

Non-glucose secretagogues were next used. Potassium chloride (KCl) treatment at 30 mM induced strong and equivalent insulin secretion in both LP and HP cells, demonstrating that HP cells were still able to secrete insulin. KCl stimulated insulin secretion in HP was significantly greater than with 25 mM glucose (p<0.05, Figure 1C). L-arginine, is a potentiator of GSIS, was effective in both cells (p<0.05, Figure 1C) but HP cells did not achieve levels near those in LP cells or with KCl stimulation.

### Intracellular ATP Content in Low and High Passage MIN6 Cells

**Figure 2:**
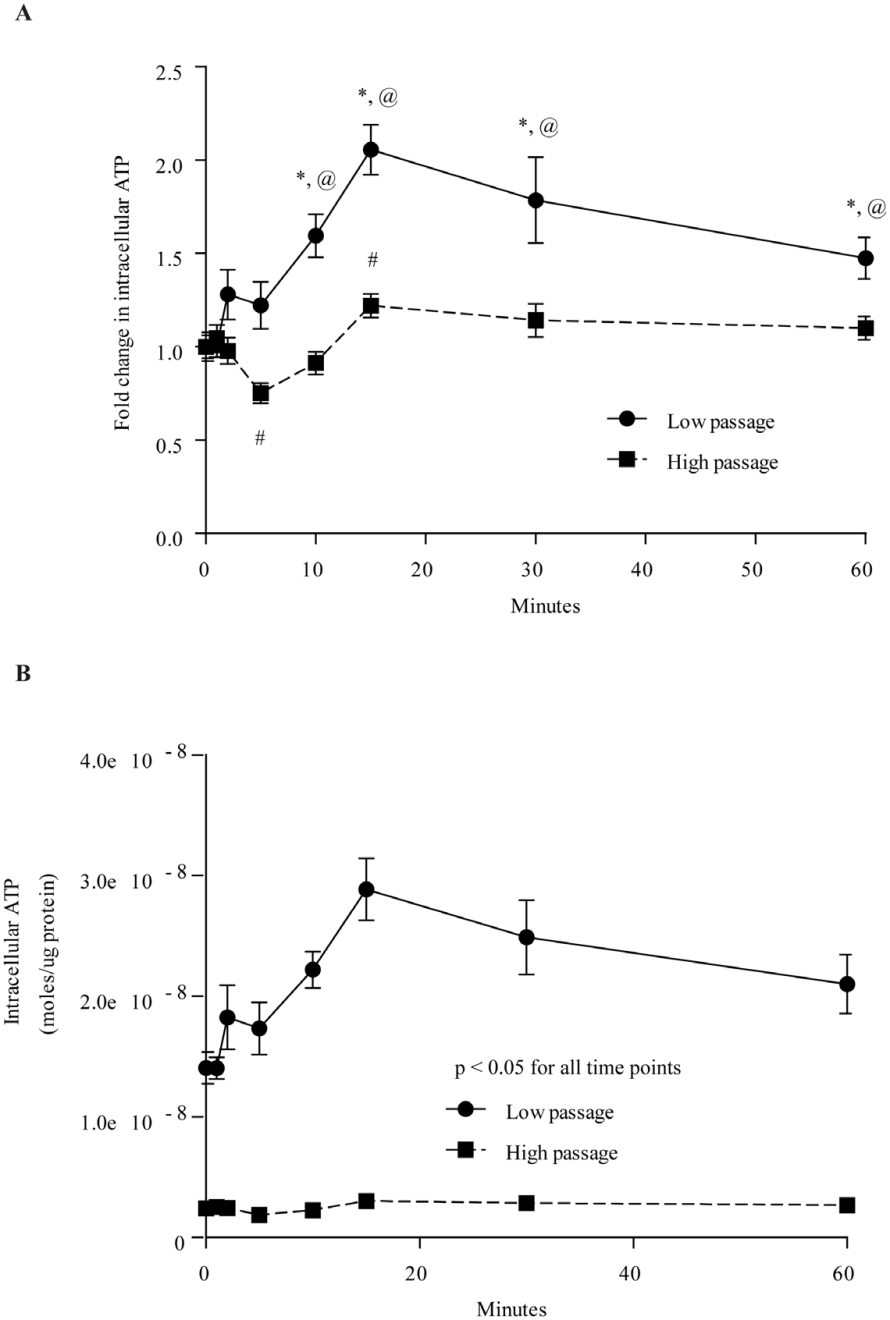
Intracellular ATP content in LP and HP MIN6 cells. A) Time course showing the fold change in intracellular ATP content. HP MIN6 cells only exhibited significant fold change differences from basal ATP content at 2 and 15 minutes after glucose stimulation. LP MIN6 cells had significantly higher fold changes from basal after 10 minutes of glucose stimulation. B) Time course showing the intracellular ATP molar concentration. HP MIN6 cells had significantly less intracellular ATP content at all time points. Error bars are ± SEM and n = 6. * p<0.05 compared to LP MIN6 cells at basal, # p<0.05 compared to HP MIN6 cells at basal, @ p<0.05 LP vs. HP MIN6 cells at their respective time points.

LP cells responded to 25 mM glucose with increased ATP content, resulting in significant increases over basal levels from 10 minutes onwards (p<0.05, Figure 2A). This was not observed in HP cells, with the only significant increase at 15 minutes (Figure 2A). Figure 2A shows the intracellular ATP content expressed as a fold change compared with their respective basal levels. Surprisingly, there was a significant decrease in ATP content at 5 minutes in HP cells, which was not seen in LP cells (p<0.05). Figure 2B shows the ATP data expressed as raw numbers. LP cells had at least 5-fold higher ATP at all time points compared to HP cells (p<0.01).

### Morphology and Cell Cycle Progression

**Figure 3:**
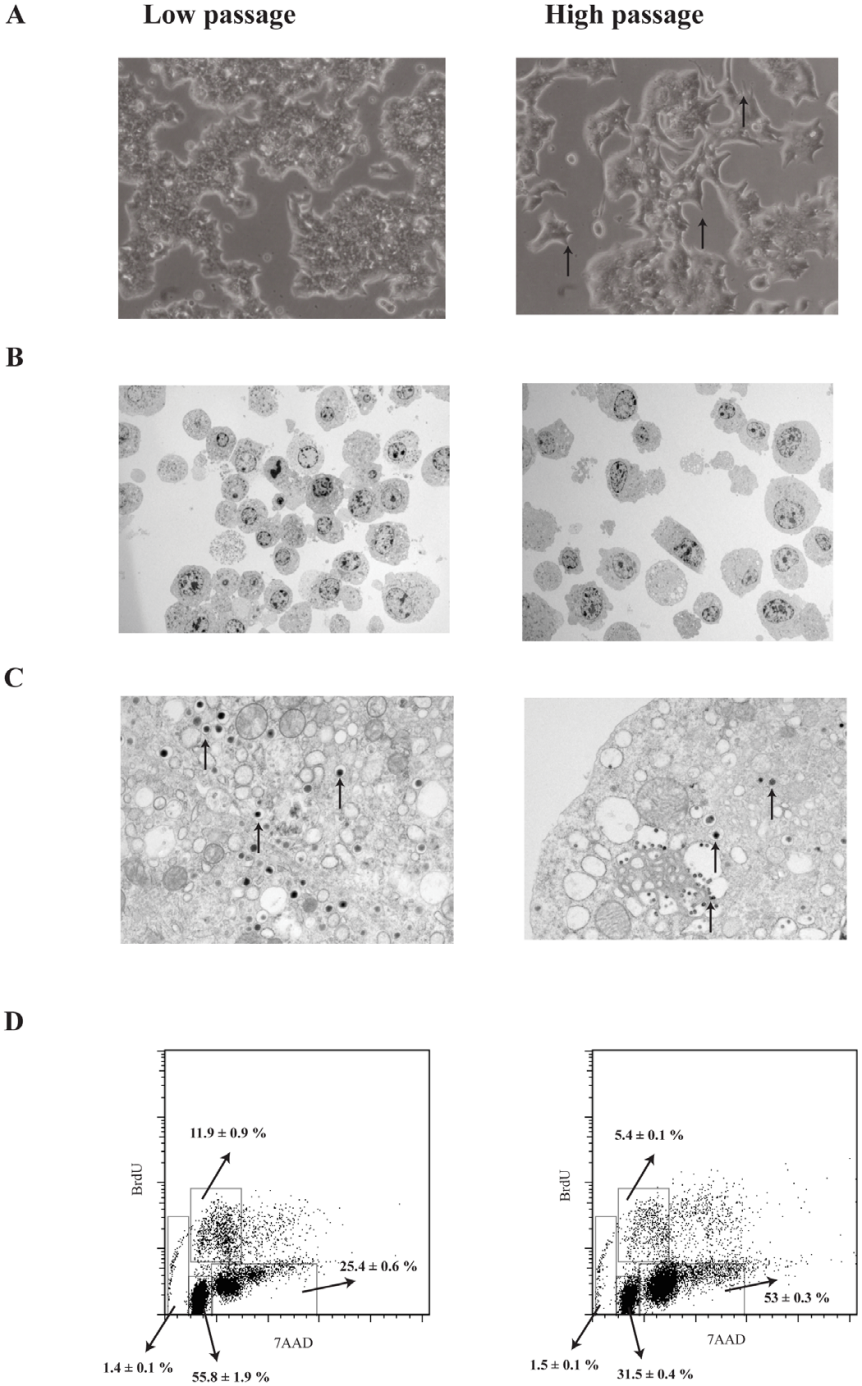
Morphology and cell proliferation in LP and HP MIN6 cells. A) Pointed formations (as indicated by the arrows) were observed in HP MIN6 cells and appeared more irregular than LP MIN6 cells. B) Electron microscope pictures of LP and HP MIN6 cells under low magnification. HP MIN6 cells were larger compared to LP MIN6 cells (200.56 µM^2^ versus 111.89 µM^2^, p<0.05). C) LP MIN6 cells displayed distribution and frequency of insulin granules (as indicated by the arrows) typical of an islet cell while HP MIN6 cells had slightly less insulin granules. D) Representative FACS plot of BrdU and 7AAD staining. HP MIN6 cells had a lower percentage of cells in the S phase of DNA replication (5.4% versus 11.9%, p<0.05). Errors values are ± SEM and n = 3.

Morphologically, LP MIN6 cells generally appeared round whereas the HP cells had a more irregular shape with pointed protrusions (Figure 3A). Cells were viewed under an electron microscope. HP cells were larger with an average size of 200.56 µM^2^ compared to an average size of 111.89 µM^2^ in LP MIN6 cells (p<0.05, Figure 3B). The distribution and frequency of insulin granules in LP MIN6 cells were typical. However, there were reduced insulin granules per field of view in HP cells (Figure 3C, representative insulin granules are indicated with an arrow).

To determine whether HP MIN6 cells might be consuming ATP due to increased cell proliferation, flow cytometry analysis of BrdU incorporation and 7AAD staining in was performed. HP cells had a lower percentage of cells in S phase of DNA replication (5.4% versus 11.9%, p<0.001, Figure 3D). This suggests that HP cells were not utilizing ATP by more rapid cell cycling.

### Glucose Oxidation, Glucose Uptake, and Lipid Oxidation

**Figure 4:**
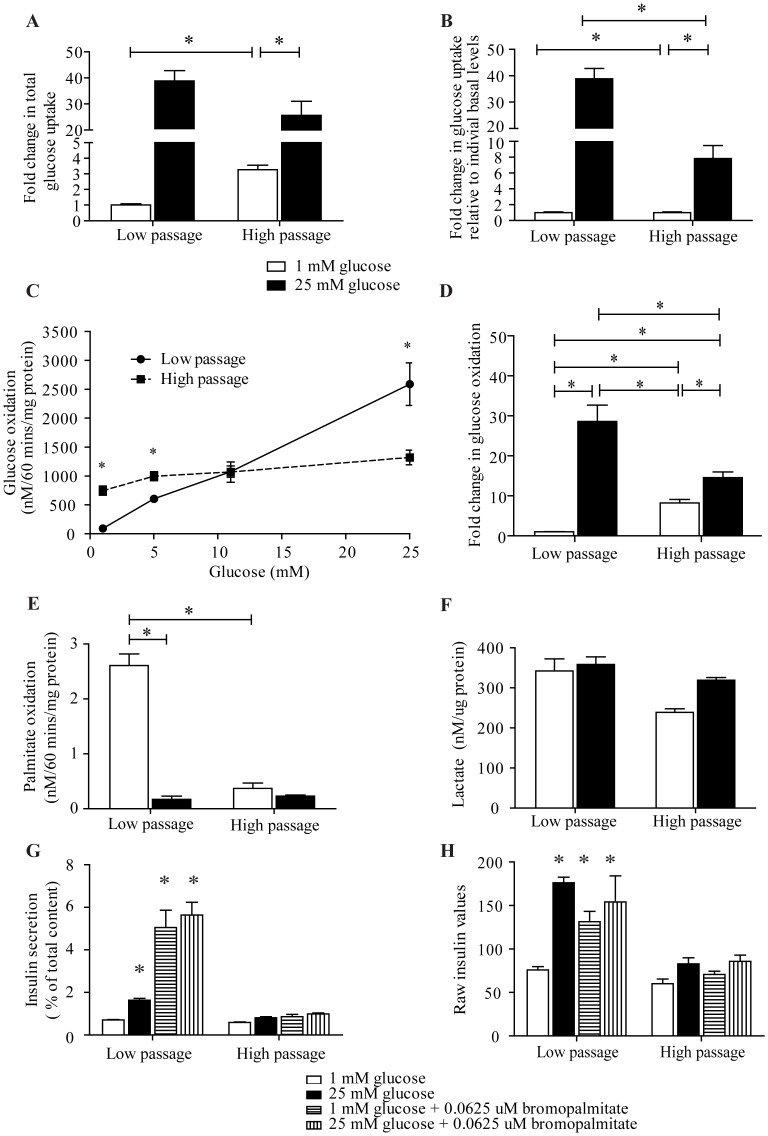
Glucose uptake, glucose oxidation, and lipid oxidation in LP and HP MIN6 cells. A) Both LP and HP MIN6 cells had a significant increase in total glucose uptake from 1 to 25 mM glucose when compared to 1 mM glucose uptake in LP cells. B) When fold change in glucose uptake is compared to their respective basal levels, HP cells only had an approximate 7-fold increase compared to an approximate 38-fold increase in LP cells. C) LP MIN6 cells exhibited a dose response in glucose oxidation when stimulated with increasing amounts of glucose. HP MIN6 cells did not show this response, with an 8-fold increase in glucose oxidation at 1 mM glucose and only increasing by 1.8-fold at 25 mM glucose. D) Glucose oxidation in low and high passage MIN6 cells expressed as the fold change compared to LP MIN6 cells at 1 mM glucose. E) HP MIN6 showed no preferential lipid oxidation at low glucose concentrations as that seen in LP MIN6 cells. There were no significant differences in lipid oxidation between 1 and 25 mM glucose in HP MIN6 cells. F) There was a significant increase in lactate from 1 to 25 mM glucose. G) 0.0625 µM bromopalmitate treatment for 2 hours would seem to increase insulin secretion but total insulin values content was low indicating cell death. H) Raw insulin values showing reduced insulin secretion with bromopalmitate treatment in both LP and HP MIN6 cells. Error bars are ± SEM and n = 6. * p<0.05.

Glucose uptake was measured using the glucose analogue 2-deoxy-[1,2-^3^H]-glucose. In cells where there is active glucose uptake, such as differentiated C2C12 myotubes, there is ∼50% decrease in 2-deoxy-[1,2-^3^H]-glucose uptake from basal to high glucose conditions, due to dilution by ‘cold’ non-radioactive glucose [20]. Figure 4A shows an estimation of total glucose uptake in the cells (i.e. including calculated uptake of non-radioactive glucose uptake plus 2-deoxy-[1,2-^3^H]-glucose). Interestingly, HP cells had approximately 3-fold higher 2-deoxy-[1,2-^3^H]-glucose uptake at basal glucose versus LP cells (p<0.01) but decreased uptake at 25 mM glucose suggesting that their uptake was at a greater proportion of maximal capacity at baseline (Figure 4A). Both low and HP cells had a significant increase in calculated total glucose (radioactive plus non radioactive) uptake from 1 to 25 mM glucose which was ∼50% greater in LP cells, although not significant (Figure 4A). However, Figure 4B shows the fold-change in total glucose uptake compared to their respective basal levels. LP cells had ∼38-fold increase from 1 to 25 mM glucose whereas HP cells only had ∼7-fold change (p<0.05).

Glucose oxidation was measured by the amount of ^14^CO_2_ production from the breakdown of D-[U-^14^C]-glucose. LP cells exhibited a dose dependent increase in glucose oxidation with ∼28-fold increase from 1 to 25 mM glucose (p<0.001). Consistent with their increased basal glucose uptake, HP cells had an 8-fold higher basal glucose oxidation (p<0.001). However, they only displayed a 1.8-fold increase in glucose oxidation at 25 mM glucose. This resulted in 50% lower absolute glucose oxidation in high versus LP MIN6 cells (Figure 4C). Figure 4D shows the fold changes in glucose oxidation at 1 and 25 mM glucose.

Lipid oxidation provides an important source of energy to β-cells. It was measured by ^14^CO_2_ produced from the breakdown of [1-^14^C]-palmitic acid. In LP cells, lipid oxidation was high at 1 mM glucose and as expected [21], was markedly reduced at 25 mM glucose by ∼6-fold (p<0.0001, Figure 4E). In contrast, basal lipid oxidation in HP MIN6 cells was low (p<0.0001) and there was no significant change at 25 mM glucose (Figure 4E).

To determine whether the loss of basal lipid oxidation was important for the phenotype of HP MIN6 cells, bromopalmitate was used to inhibit lipid oxidation. Higher concentrations of bromopalmitate caused rapid cell death, indicating the importance of lipid oxidation (data not shown). There was an indication of increased insulin secretion with the addition of 0.0625 µM bromopalmitate for 2 hours (Figure 4G), however, we think this is still due to toxicity as the raw data without correction for total insulin content negates the increase (Figure 4H).

### Lactate

**Figure 5:**
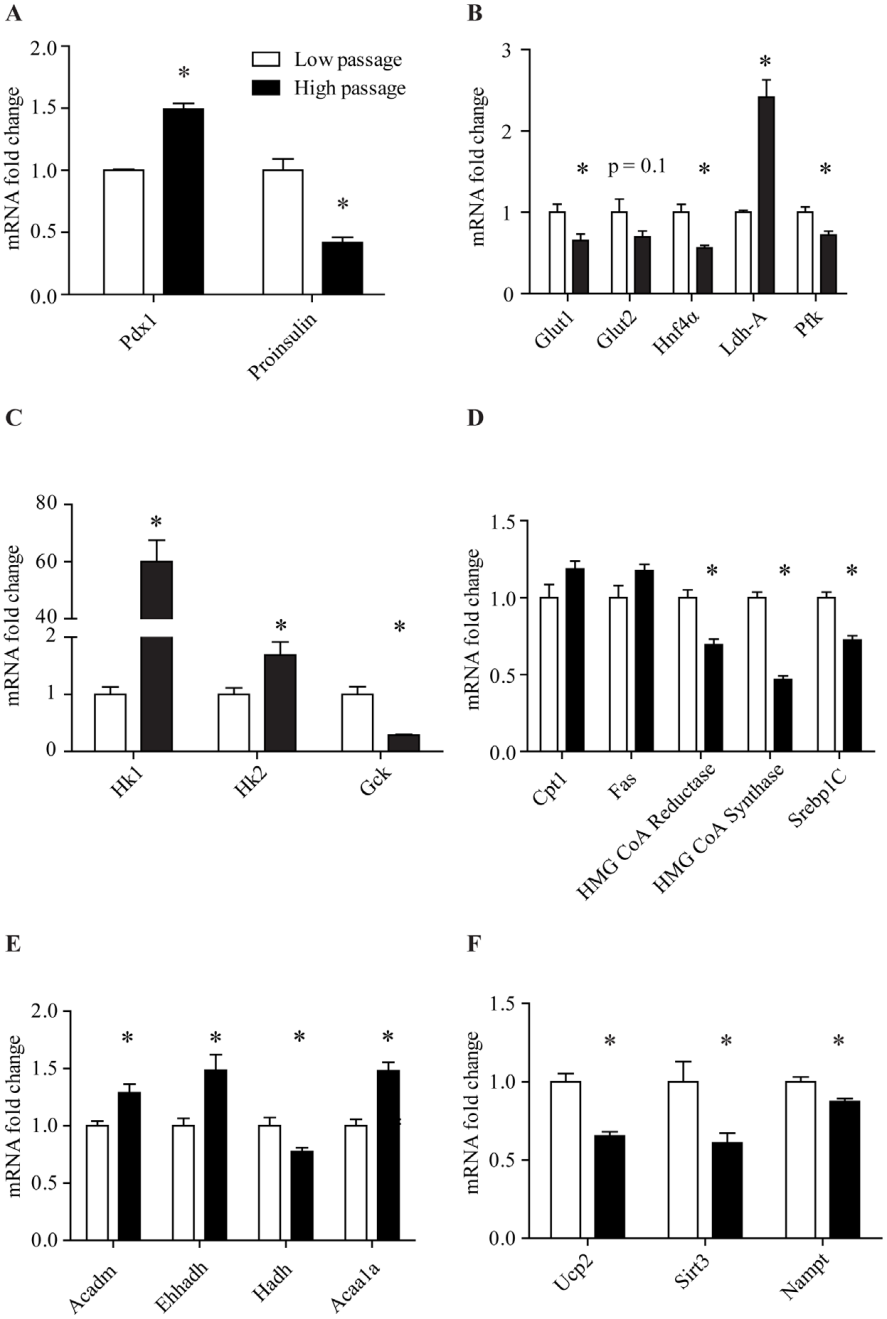
Gene expression in HP and LP MIN6 cells. A) HP MIN6 cells had increased expression of *Pdx1* and reduced expression of *proinsulin*. B) HP MIN6 cells had reduced expressions of the glucose transporter *Glut1* and the key glycolytic gene *Pfk*. There was a greater than 2-fold increase in the glycolytic gene *Ldh-A* in high passage MIN6 cells. C) HP MIN6 cells had increased expressions of *Hk1* and *Hk2*, accompanied with a decrease in *Gck*. D) HP MIN6 cells also had reduced expressions of cholesterol synthesizing genes *HMG CoA reductase* and *HMG CoA synthase*, and the transcription factor for lipid synthesis *Srebp1C*. E) HP MIN6 cells had increased expression of lipid oxidation genes *Acadm, Ehhadh, Acaa1a* and increased *Hadh* expression. F) HP MIN6 cells had reduced *Ucp2*, *Sirt3*, and *Nampt* expression. Error bars are ± SEM and n = 3.

To determine if the alterations in glucose metabolism in HP MIN6 cells also involved in increased shunting of glucose into lactate synthesis rather than oxidative metabolism, we measured expression of *Ldh-A* and lactate. No significant differences in lactate were observed between 1 and 25 mM glucose in LP MIN6 cells (Figure 4F). HP cells had a non-significant decrease at 1 mM glucose and a significant increase at 25 mM glucose (Figure 4F). This was still lower than the LP cells. HP MIN6 cells had a 2-fold increase in *Ldh-A* expression (p<0.00001, Figure 5A).

### Gene Expression between Low and High Passage MIN6 Cells

The mRNA expression of the pancreatic transcription factor *pancreatic duodenal homeobox gene-1* (*Pdx1*) and *proinsulin* were examined. HP MIN6 cells had ∼50% increase in *Pdx1* expression (p<0.00001) and ∼60% decrease in *proinsulin* expression (p<0.00001, Figure 5A). HP MIN6 cells had reduced expression of mRNAs encoding the glucose transporter *Glut1*, *Hnf4α*, and *phosphofructokinase* (*Pfk*, p<0.05, Figure 5B). HP MIN6 cells had increased expressions of *hexokinase (Hk) 1* and *2*, with *Hk1* increased approximately 60-fold (p<0.01, Figure 5C). The key glycolytic gene *glucokinase* (*Gck,* also known as hexokinase IV) was decreased in HP cells (p<0.01, Figure 5C).

Analysis of lipid handling genes revealed that HP cells had reduced expression of the important lipid synthesis transcription factor *Srebp1c* (p<0.0001, Figure 5D) and decreased cholesterol synthesizing genes *HMG CoA reductase* and *HMG CoA synthase*. HP cells had lower expression of *L-β-hydroxyacyl coA dehydrogenase (Hadh)* and higher *acyl-coA dehydrogenase medium chain (Acadm), enoyl coA hydrotase (Ehhadh),* and *acetyl-coA acyltransferase 1a (Acaa1a)* (Figure 5E).

The mitochondrial uncoupling gene, *Ucp2*, was decreased by 35% in HP MIN6 cells (p<0.0001, Figure 5F). Only very low levels of *Ucp1* mRNA were present in both LP and HP cells and did not differ (data not shown). *Nampt* has been reported in other tissues to regulate *Sirt3* [22] and *Sirt3* has been shown to regulate lipid oxidation [23]. Expression of both *Nampt* and *Sirt3* was significantly decreased in HP MIN6 cells (Figure 5F).

## Discussion

Subjects with T2D lose first phase insulin secretion early in the natural history of disease progression [9], [24]–[26]. In this study, we used MIN6 cells to examine defects in first phase insulin secretion. With high passage these cells had complete loss of first phase insulin secretion and an overall impairment in GSIS. Previous studies in LP and HP MIN6 cells have shown both expression and protein level changes [12]–[14]; however this is the first report to profile metabolic changes in these cells. HIT-T15 cells, another clonal beta-cell type, are also known to lose both first and second phase GSIS with increased passage. This was attributed to decreased insulin gene expression and insulin content and was suggested to be related to constant exposure to 11 mM glucose [27]. Our HP MIN6 cells displayed similar characteristics, with reduced insulin granule formation and *proinsulin* mRNA expression.

HP cells responded normally to KCl, which works by closing the K_ATP_ channels, downstream of glucose metabolism. Normal KCl-stimulated insulin secretion clearly indicated that the defects in first phase insulin secretion are not in insulin synthesis or secretion capability but lie upstream. L-arginine stimulation of insulin secretion was retained in HP MIN6 cells, but to a lesser extent than LP cells. L-arginine stimulates insulin by inducing Ca^2+^ release from mitochondria via the actions of nitrogen oxides in the presence of glucose [28], [29]. These changes again suggest that the defects lie upstream of the K_ATP_ channels.

The lack of an increase in early insulin secretion in HP MIN6 cells was consistent with the failure of increase in intracellular ATP concentration with glucose stimulation. Increased ATP:ADP ratio is crucial in beta-cells as this precedes a cascade of steps necessary for insulin secretion [2]–[4]. HP MIN6 cells had significantly decreased intracellular ATP at 5 minutes after glucose stimulation but the mechanism or the significance of this is unknown. It is possible that ATP consuming events such as glycolysis could be increased shortly after glucose stimulation without subsequent ATP generation but this needs to be verified. However, impaired high-glucose stimulated ATP was ultimately a result of decreased glucose uptake and impaired glucose oxidation. This was associated with a significant decrease in *Glut1* and a trend to decreased *Glut2*. Glut2 is the main glucose sensor in the rodent beta-cell due to its high *K_m_* [30]. There was also a reduction in the rate limiting glycolytic enzymes *Gck* and *Pfk*, both of which are important in the provision of substrates to the TCA cycle and oxidative phosphorylation.

Mitochondrial oxidative phosphorylation plays an important role for insulin secretion, as it provides much of the needed ATP to change the ATP:ADP ratio [31]–[33]. The question of the relative contribution of mitochondrial oxidative phosphorylation versus other pathways in physiological insulin secretion remains controversial [34]–[37]. Regardless, in order for oxidative phosphorylation to be able to generate ATP, substrates must be provided to it by the glycolytic pathway and the TCA cycle. This flow of substrates could be inhibited by high *Ldh-A* expression. Ldh-A converts pyruvate to lactate and NAD^+^ and thus decreases pyruvate. Over-expression of *Ldh-A* in MIN6 cells has been shown to attenuate GSIS [38]. However, lactate in HP MIN6 cells was actually decreased, suggesting that this was not a mechanistic change. There was no change in oxygen respiration rates as measured by a Clark-type oxygen electrode (data not shown).

Some lipid synthesis genes were also down-regulated in HP MIN6 cells, including the important transcription factor *Srebp1c*. Lipids, as well as glucose, are a major source of ATP. Per Mole of substrate, lipid yields more ATP than glucose with up to 136 molecules of ATP generated per palmitate molecule versus 30–36 ATP per glucose molecule. Rough calculations estimating the amount of ATP able to be produced from glucose and lipid oxidation indicate that HP MIN6 cells produce more intracellular ATP compared to LP MIN6 cells at 1 mM glucose. This suggests that HP MIN6 cells are either utilizing much more ATP at 1mM glucose or they are wasting energy. HP MIN6 cells, morphologically different to LP MIN6 cells, were not utilizing extra energy in increased cell proliferation as they had a slower rate of cell division, as evidenced by FACS analysis of the BrdU uptake.

The uncoupling proteins function by dissipating the energy from glucose/lipid oxidation as heat rather than flowing through the electron transport chain to produce ATP [39]. The uncoupling protein Ucp1 is the classic and most well known UCP out of five (Ucp1– Ucp5) but is predominantly expressed in brown adipose tissue [40], [41]. There were very low levels of *Ucp1* expression in both low and HP MIN6 cells and these were not significantly different. We measured *Ucp2* expression as this has been proposed to be major factor in obesity, beta cell dysfunction, and type 2 diabetes, negatively regulating insulin secretion [42]. Recent findings have proposed that Ucp2 does not act as an uncoupler and does not contribute to adaptive thermogenesis [41], [43]–[45]. In fact, a previous study has shown that a decrease in intracellular ATP content down-regulated *Ucp2* expression in mouse hepatocytes [46] and mice with homozygous knockout of *Ucp2* have impaired beta cell function, possibly due to increased oxidative stress [47]. Interestingly, HP MIN6 cells had a 35% lower expression in *Ucp2*. The decreased *Ucp2* in HP MIN6 may be secondary to the markedly decreased ATP content.

Recent findings have indicated the enzyme nicotinamide phospho-ribosyl-transferase (Nampt) and nicotinamide adenine dinucleotide (NAD) biosynthesis in insulin secretion and metabolism [48], [49]. The enzyme Nampt is the rate limiting step in NAD biosynthesis and mice with a heterozygous deletion of *Nampt* have been shown to have impaired glucose tolerance and isolated islets have reduced GSIS [49]. There is also reduced expression of Sirt3 in streptozotocin induced diabetic mice [50]. The sirtuins are a family of deactetylases and mono-ADP-ribosyltransferases, of which there are seven in mammals, that use NAD as a substrate [51]. It has been previously reported that Sirt1 regulates insulin secretion by repressing *Ucp2* in β-cells [52] but there are no reports regarding Sirt3 and beta cell function. HP MIN6 cells had significantly reduced expression of both *Nampt* and *Sirt3*.

Sirt3 regulates mitochondrial fatty acid oxidation in other tissues and *Sirt3^−/−^* mice have reduced ATP levels in various tissues [23], [53]. HP MIN6 cells had significantly reduced *Sirt3* expression and reduced lipid oxidation, suggesting that decreased Sirt3 may be driving this.

As lipid oxidation at 1mM glucose failed to provide the cell with ATP, we believe that HP MIN6 cells attempted to compensate for this by increasing glucose uptake and oxidation in the basal state. To determine the importance of basal lipid oxidation, it was blocked with the non-metabolizable lipid bromopalmitate, but this caused cell death and further reductions in insulin secretion.

This data shows that HP MIN6 cells have a very different metabolic profile compared to LP cells. A higher proportion of the HP MIN6 cells had aneuploidy (data not shown) which in other cell lines is correlated with genetic instability [54], [55]. Clearly, some of these metabolic changes are causative and some are compensatory in response to a failure in increasing intracellular ATP. It is interesting to note that many of these changes also occur in patients with T2D - impaired insulin response to L-arginine [56], decreased islet glucose oxidation [57], increased whole body glucose uptake at basal glucose levels [58], decreased *Gck*, *Hnf4α*, and *Pfk*, and increased *Pdx1* expression [19], [57].

**Figure 6:**
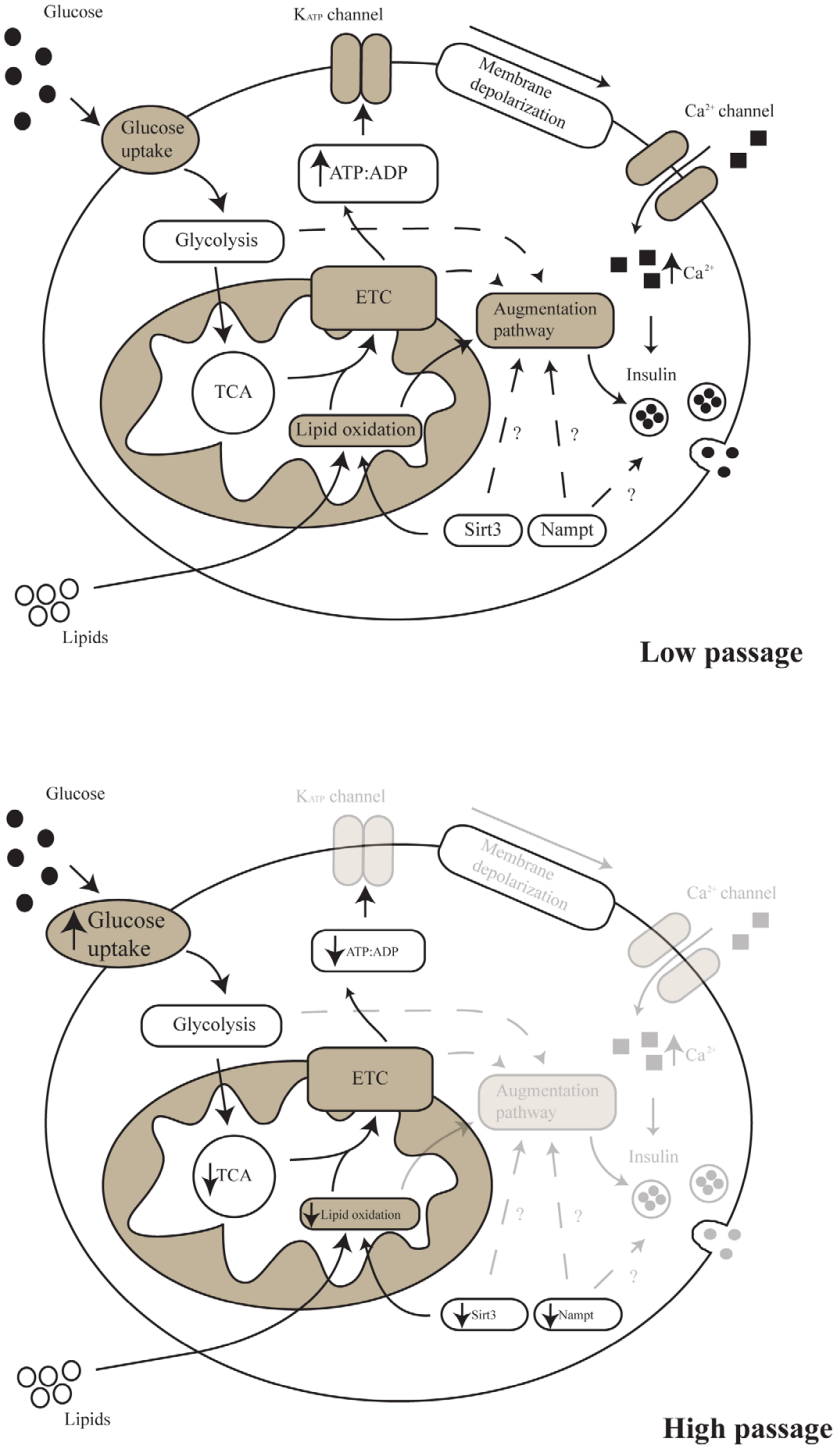
Model of insulin secretion in LP and HP MIN6 cells. HP MIN6 cells increased glucose uptake at 1 mM glucose and decreased expression of some glycolytic genes. Glucose oxidation is decreased compared to LP MIN6 cells when stimulated with glucose, leading to reduced intracellular ATP content and impaired insulin secretion. Lipid oxidation was also reduced. Reduced *Sirt3* and *Nampt* gene expression may be responsible for the impaired metabolism of glucose, ultimately leading to reduced insulin secretion.

We hypothesize that reduced *Sirt3* and *Nampt* expression are a potential mechanism underlying these metabolic changes, contributing to the decreased lipid oxidation and decreased *Glut1* and some glycolytic gene expression. This led to impaired glucose uptake and a decrease in glucose oxidation (Figure 6).

## References

[1] Curry DL, Bennett LL, Grodsky GM (1968). Dynamics of insulin secretion by the perfused rat pancreas.. Endocrinology.

[2] Henquin JC (2000). Triggering and amplifying pathways of regulation of insulin secretion by glucose.. Diabetes.

[3] Newgard CB, McGarry JD (1995). Metabolic coupling factors in pancreatic beta-cell signal transduction.. Annu Rev Biochem.

[4] Straub SG, Sharp GW (2002). Glucose-stimulated signaling pathways in biphasic insulin secretion.. Diabetes Metab Res Rev.

[5] Ashcroft FM, Harrison DE, Ashcroft SJ (1984). Glucose induces closure of single potassium channels in isolated rat pancreatic beta-cells.. Nature.

[6] Cook DL, Hales CN (1984). Intracellular ATP directly blocks K+ channels in pancreatic B-cells.. Nature.

[7] Kelly RP, Sutton R, Ashcroft FM (1991). Voltage-activated calcium and potassium currents in human pancreatic beta-cells.. J Physiol.

[8] Davies MJ, Rayman G, Grenfell A, Gray IP, Day JL (1994). Loss of the first phase insulin response to intravenous glucose in subjects with persistent impaired glucose tolerance.. Diabet Med.

[9] Pratley RE, Weyer C (2001). The role of impaired early insulin secretion in the pathogenesis of Type II diabetes mellitus.. Diabetologia.

[10] Ishihara H, Asano T, Tsukuda K, Katagiri H, Inukai K (1993). Pancreatic beta cell line MIN6 exhibits characteristics of glucose metabolism and glucose-stimulated insulin secretion similar to those of normal islets.. Diabetologia.

[11] Miyazaki J, Araki K, Yamato E, Ikegami H, Asano T (1990). Establishment of a pancreatic beta cell line that retains glucose-inducible insulin secretion: special reference to expression of glucose transporter isoforms.. Endocrinology.

[12] Dowling P, O’Driscoll L, O’Sullivan F, Dowd A, Henry M (2006). Proteomic screening of glucose-responsive and glucose non-responsive MIN-6 beta cells reveals differential expression of proteins involved in protein folding, secretion and oxidative stress.. Proteomics.

[13] O’Driscoll L, Gammell P, Clynes M (2004). Mechanisms associated with loss of glucose responsiveness in beta cells.. Transplant Proc.

[14] O’Driscoll L, Gammell P, McKiernan E, Ryan E, Jeppesen PB (2006). Phenotypic and global gene expression profile changes between low passage and high passage MIN-6 cells.. J Endocrinol.

[15] Lilla V, Webb G, Rickenbach K, Maturana A, Steiner DF (2003). Differential gene expression in well-regulated and dysregulated pancreatic beta-cell (MIN6) sublines.. Endocrinology.

[16] Minami K, Yano H, Miki T, Nagashima K, Wang CZ (2000). Insulin secretion and differential gene expression in glucose-responsive and -unresponsive MIN6 sublines.. Am J Physiol Endocrinol Metab.

[17] Akerfeldt MC, Laybutt DR (2011). Inhibition of Id1 augments insulin secretion and protects against high-fat diet-induced glucose intolerance.. Diabetes.

[18] Cheng K, Ho K, Stokes R, Scott C, Lau SM (2010). Hypoxia-inducible factor-1alpha regulates beta cell function in mouse and human islets.. J Clin Invest.

[19] Gunton JE, Kulkarni RN, Yim S, Okada T, Hawthorne WJ (2005). Loss of ARNT/HIF1beta mediates altered gene expression and pancreatic-islet dysfunction in human type 2 diabetes.. Cell.

[20] Nedachi T, Kanzaki M (2006). Regulation of glucose transporters by insulin and extracellular glucose in C2C12 myotubes.. Am J Physiol Endocrinol Metab.

[21] Corkey BE, Glennon MC, Chen KS, Deeney JT, Matschinsky FM (1989). A role for malonyl-CoA in glucose-stimulated insulin secretion from clonal pancreatic beta-cells.. J Biol Chem.

[22] Yang H, Yang T, Baur JA, Perez E, Matsui T (2007). Nutrient-sensitive mitochondrial NAD+ levels dictate cell survival.. Cell.

[23] Hirschey MD, Shimazu T, Goetzman E, Jing E, Schwer B (2010). SIRT3 regulates mitochondrial fatty-acid oxidation by reversible enzyme deacetylation.. Nature.

[24] Bagdade JD, Bierman EL, Porte D (1967). The significance of basal insulin levels in the evaluation of the insulin response to glucose in diabetic and nondiabetic subjects.. J Clin Invest.

[25] Butler AE, Janson J, Bonner-Weir S, Ritzel R, Rizza RA (2003). Beta-cell deficit and increased beta-cell apoptosis in humans with type 2 diabetes.. Diabetes.

[26] Gerich JE (1998). The genetic basis of type 2 diabetes mellitus: impaired insulin secretion versus impaired insulin sensitivity.. Endocr Rev.

[27] Robertson RP, Zhang HJ, Pyzdrowski KL, Walseth TF (1992). Preservation of insulin mRNA levels and insulin secretion in HIT cells by avoidance of chronic exposure to high glucose concentrations.. J Clin Invest.

[28] Laffranchi R, Gogvadze V, Richter C, Spinas GA (1995). Nitric oxide (nitrogen monoxide, NO) stimulates insulin secretion by inducing calcium release from mitochondria.. Biochem Biophys Res Commun.

[29] Schmidt HH, Warner TD, Ishii K, Sheng H, Murad F (1992). Insulin secretion from pancreatic B cells caused by L-arginine-derived nitrogen oxides.. Science.

[30] Efrat S, Tal M, Lodish HF (1994). The pancreatic beta-cell glucose sensor.. Trends Biochem Sci.

[31] Liang Y, Bai G, Doliba N, Buettger C, Wang L (1996). Glucose metabolism and insulin release in mouse beta HC9 cells, as model for wild-type pancreatic beta-cells.. Am J Physiol.

[32] Maechler P, Kennedy ED, Pozzan T, Wollheim CB (1997). Mitochondrial activation directly triggers the exocytosis of insulin in permeabilized pancreatic beta-cells.. Embo J.

[33] Sekine N, Cirulli V, Regazzi R, Brown LJ, Gine E (1994). Low lactate dehydrogenase and high mitochondrial glycerol phosphate dehydrogenase in pancreatic beta-cells. Potential role in nutrient sensing.. J Biol Chem.

[34] Matschinsky FM, Ellerman J (1973). Dissociation of the insulin releasing and the metabolic functions of hexoses in islets of Langerhans.. Biochem Biophys Res Commun.

[35] Mertz RJ, Worley JF, Spencer B, Johnson JH, Dukes ID (1996). Activation of stimulus-secretion coupling in pancreatic beta-cells by specific products of glucose metabolism. Evidence for privileged signaling by glycolysis.. J Biol Chem.

[36] Sener A, Kawazu S, Hutton JC, Boschero AC, Devis G (1978). The stimulus-secretion coupling of glucose-induced insulin release. Effect of exogenous pyruvate on islet function.. Biochem J.

[37] Sener A, Malaisse WJ (1987). Stimulation by D-glucose of mitochondrial oxidative events in islet cells.. Biochem J.

[38] Zhao C, Rutter GA (1998). Overexpression of lactate dehydrogenase A attenuates glucose-induced insulin secretion in stable MIN-6 beta-cell lines.. FEBS Lett.

[39] Andrews ZB, Diano S, Horvath TL (2005). Mitochondrial uncoupling proteins in the CNS: in support of function and survival.. Nat Rev Neurosci.

[40] Klingenberg M, Echtay KS (2001). Uncoupling proteins: the issues from a biochemist point of view.. Biochim Biophys Acta.

[41] Rousset S, Alves-Guerra MC, Mozo J, Miroux B, Cassard-Doulcier AM (2004). The biology of mitochondrial uncoupling proteins.. Diabetes.

[42] Zhang CY, Baffy G, Perret P, Krauss S, Peroni O (2001). Uncoupling protein-2 negatively regulates insulin secretion and is a major link between obesity, beta cell dysfunction, and type 2 diabetes.. Cell.

[43] Brand MD, Esteves TC (2005). Physiological functions of the mitochondrial uncoupling proteins UCP2 and UCP3.. Cell Metab.

[44] Krauss S, Zhang CY, Lowell BB (2002). A significant portion of mitochondrial proton leak in intact thymocytes depends on expression of UCP2.. Proc Natl Acad Sci U S A.

[45] Pi J, Collins S (2010). Reactive oxygen species and uncoupling protein 2 in pancreatic beta-cell function.. Diabetes Obes Metab.

[46] Cheng G, Polito CC, Haines JK, Shafizadeh SF, Fiorini RN (2003). Decrease of intracellular ATP content downregulated UCP2 expression in mouse hepatocytes.. Biochem Biophys Res Commun.

[47] Pi J, Bai Y, Daniel KW, Liu D, Lyght O (2009). Persistent oxidative stress due to absence of uncoupling protein 2 associated with impaired pancreatic beta-cell function.. Endocrinology.

[48] Garten A, Petzold S, Korner A, Imai S, Kiess W (2009). Nampt: linking NAD biology, metabolism and cancer.. Trends Endocrinol Metab.

[49] Revollo JR, Korner A, Mills KF, Satoh A, Wang T (2007). Nampt/PBEF/Visfatin regulates insulin secretion in beta cells as a systemic NAD biosynthetic enzyme.. Cell Metab.

[50] Yechoor VK, Patti ME, Ueki K, Laustsen PG, Saccone R (2004). Distinct pathways of insulin-regulated versus diabetes-regulated gene expression: an in vivo analysis in MIRKO mice.. Proc Natl Acad Sci U S A.

[51] Guarente L, Picard F (2005). Calorie restriction–the SIR2 connection.. Cell.

[52] Bordone L, Motta MC, Picard F, Robinson A, Jhala US (2006). Sirt1 regulates insulin secretion by repressing UCP2 in pancreatic beta cells.. PLoS Biol.

[53] Ahn BH, Kim HS, Song S, Lee IH, Liu J (2008). A role for the mitochondrial deacetylase Sirt3 in regulating energy homeostasis.. Proc Natl Acad Sci U S A.

[54] Ghadimi BM, Sackett DL, Difilippantonio MJ, Schrock E, Neumann T (2000). Centrosome amplification and instability occurs exclusively in aneuploid, but not in diploid colorectal cancer cell lines, and correlates with numerical chromosomal aberrations.. Genes Chromosomes Cancer.

[55] Duesberg P, Rausch C, Rasnick D, Hehlmann R (1998). Genetic instability of cancer cells is proportional to their degree of aneuploidy.. Proc Natl Acad Sci U S A.

[56] Ward WK, Bolgiano DC, McKnight B, Halter JB, Porte D (1984). Diminished B cell secretory capacity in patients with noninsulin-dependent diabetes mellitus.. J Clin Invest.

[57] Del Guerra S, Lupi R, Marselli L, Masini M, Bugliani M (2005). Functional and molecular defects of pancreatic islets in human type 2 diabetes.. Diabetes.

[58] Thorburn AW, Gumbiner B, Bulacan F, Wallace P, Henry RR (1990). Intracellular glucose oxidation and glycogen synthase activity are reduced in non-insulin-dependent (type II) diabetes independent of impaired glucose uptake.. J Clin Invest.

